# Evaluation of Retinal Sensitivity in Complete Retinal-Pigment-Epithelium and Outer Retinal Atrophy (cRORA) Lesions in Intermediate Age-Related Macular Degeneration (iAMD) by High-Resolution Microperimetry

**DOI:** 10.3390/jcm13247785

**Published:** 2024-12-20

**Authors:** Marlene Saßmannshausen, Julius Ameln, Leon von der Emde, Frank G. Holz, Thomas Ach, Wolf M. Harmening

**Affiliations:** Department of Ophthalmology, University Hospital Bonn, Venusberg-Campus 1, 53127 Bonn, Germany

**Keywords:** AMD, geographic atrophy, precursor lesion, retinal function, adaptive optics, AOSLO-MP, clinical outcome measures

## Abstract

**Objective**: Lesions characterized as complete retinal pigment epithelium and outer retinal atrophy (cRORA) are linked to the progression of intermediate age-related macular degeneration (iAMD). However, the extent of functional impairment of such precursor lesions remains uncertain. **Methods**: In this cross-sectional study, 4 participants (mean age ± standard deviation: 71.5 ± 2.1 years) underwent extensive multimodal imaging and psychophysical testing of cRORA lesions secondary to iAMD. Lesion-specific functional testing was performed using patient individualized testing grids with clinical conventional available (Stimulus size: 0.43°, ~125 µm) and experimental adaptive optics scanning light ophthalmoscope (AOSLO, stimulus size 0.07°, ~20 µm) based microperimetry (MP). One cRORA lesion site and one in-eye control region were tested per patient, respectively. **Results**: AOSLO imaging revealed an overall decrease in photoreceptor reflectivity, areas of hyporeflectivity over drusen, interspersed with hyperreflective foci, and disrupted photoreceptor mosaic in regions of cRORA. Localized retinal sensitivity assessment with clinical conventional MP yielded an average loss of −14.0 ± 3.3 dB at cRORA lesions compared to the in-eye control regions. In contrast, localized visual impairment assessed by high-resolution AOSLO-MP with smaller test stimuli (20 µm) revealed a sensitivity loss of −15.1 ± 5.1 dB at cRORA lesions (*p* < 0.01). Notably, also the area surrounding cRORA lesions can be impacted. **Conclusions**: We demonstrated that cRORA lesions are associated with severe localized functional impairment. cRORA precursor lesions may thus be considered as a surrogate outcome measure in future interventional iAMD trials.

## 1. Introduction

Age-related macular degeneration (AMD) remains the leading cause for an irreversible vision loss in the elderly population within industrialized countries [1,2]. Developing effective therapies for AMD is challenging due to the slow progression of the disease, as well as regulatory requirements by agencies including the European Medicines Agency (EMA) mandating evidence for not only an effect on structural disease progression but also on functional outcomes with a direct, measurable, impact on patients’ daily lives [3,4,5,6].

Recent advances in high-resolution multimodal retinal imaging, particularly spectral domain optical coherence tomography (SD-OCT), have significantly improved the detection and characterization of precursor lesions associated with late-stage atrophic AMD. These precursor lesions, termed incomplete (iRORA) and complete (cRORA) retinal pigment epithelium and outer retinal atrophy by the international CAM group, have been identified through SD-OCT as structural markers that may precede lesions of geographic atrophy (GA) detectable also on fundus autofluorescence (FAF) imaging [7,8]. While there have been various studies proofing a direct correlation of RPE and outer retinal layer losses to visual sensitivity defects in late-stage AMD eyes with presence of GA [9,10,11,12], only few studies assessing the association of structural alterations of an early atrophy development to localized retinal sensitivity as well as the extent of retinal sensitivity losses are yet available [13,14]. At the same time, a potential discrepancy between the available test stimuli sizes and precision of clinical conventional microperimetry (MP) testing at very small structural alterations visible in OCT imaging may limit the meaningfulness of sensitivity results. Adaptive optics scanning laser ophthalmoscopy (AOSLO-) based MPapplied in Macular Telangiectasia Type 2 and Choroideremia could detect significant changes in retinal sensitivity within a just few micrometers at the border of atrophic regions [15,16].

In a previous study using AOSLO-MP and conventional MP testing, we demonstrated that iRORA lesions already exhibit a localized sensitivity loss, meeting both structural and functional criteria for a biomarker of disease progression [17]. However, it remains unclear whether cRORA is associated with even greater and more widespread localized functional impairment.

Therefore, the primary aim of this cross-sectional study is to evaluate the retinal sensitivity at cRORA lesions using experimental AOSLO-MP alongside conventional MP. The findings of this study could provide further evidence that SD-OCT imaging-based identification of cRORA represents a clinically relevant structural endpoint for future interventional trials targeting intermediate AMD (iAMD).

## 2. Methods

### 2.1. Subjects

This prospective cross-sectional study included patients with early lesions of atrophy development secondary to AMD and was conducted at the Department of Ophthalmology, University Hospital Bonn, Germany, between June and July 2024. The study has been approved by the Ethics Committee of the University of Bonn, Bonn, Germany (Nr. 125/20, approval: 7 October 2020 and Nr. 009/13, approval: 16 October 2023) and was performed according to the Tenets of the Declaration of Helsinki. Prior of conducting the study, informed written consent was obtained from all study participants after a detailed explanation of the study’s purpose, procedures, and potential consequences For study inclusion, patients were regarded as eligible in presence of large sub-RPE drusen (≥125 µm) associated with iAMD according to the Beckman classification [18] and at least one lesion of cRORA [7] as defined by the following OCT-imaging-based criteria: I: region of choroidal hypertransmission ≥ 250 µm and II: zone of attenuation/disruption of the retinal pigment epithelium (RPE) ≥ 250 µm and III: evidence of overlying photoreceptor degeneration in absence of an RPE tear. In addition, no lesions of GA defined by areas with reduced FAF signal detectable by semiautomated imaging analysis (Region Finder, Heidelberg Engineering, Heidelberg, Germany) with a lesion size exceeding 0.05 mm^2^ were allowed in study eyes [19]. Exclusion criteria included any history of other retinal diseases, glaucoma, previous history of vitreoretinal surgery, macular neovascularization and relevant anterior segment disease with media opacities. Any eyes with evidence for other retinal diseases as well as previous recorded treatment for neovascular AMD were excluded from this study. If both eyes of a study patient provided a lesion of cRORA, the eye with a more eccentric extrafoveal position of the lesion as well as better BCVA was chosen as a study eye. All patients underwent routine ophthalmological examination including best-corrected visual acuity (BCVA), slit lamp and funduscopic examination.

### 2.2. Retinal Imaging Protocol

In this study a detailed and standardized retinal imaging protocol was performed following pupil dilation (0.5% tropicamide, 2.5% phenylephrine). This imaging protocol included combined confocal scanning laser ophthalmoscopy (cSLO) and SD-OCT raster scanning (241 B-scans, 30° × 25° enhanced-depth-imaging [EDI] mode, centered on the fovea, scan distance 30 µm, automatic real-time mode 9 frames; Spectralis, Heidelberg Engineering, Heidelberg, Germany) Throughout this study, a retinal magnification factor of 291 µm/deg of visual angle was assumed.

### 2.3. SD-OCT Image Analysis for cRORA Detection

**Figure 1:**
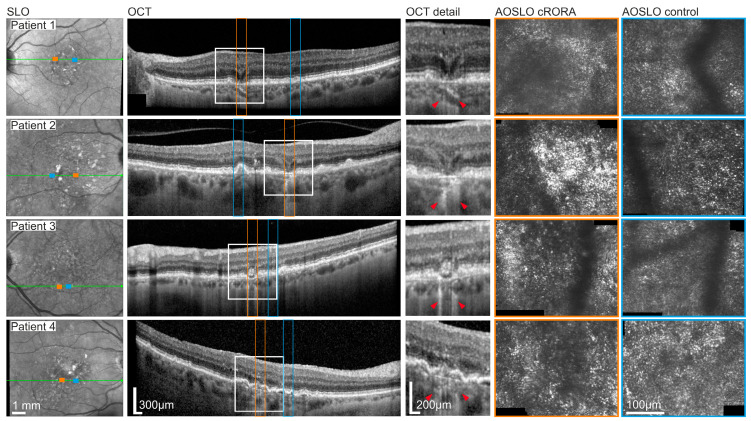
**High-resolution multimodal retinal imaging of cRORA lesions in iAMD patients.** Retinal images of four participants (rows), from left to right: Infrared SLO image, OCT B-scan and AOSLO imaging locations of cRORA lesions and in-eye control regions (orange = cRORA, blue = eccentricity-matched control) are shown. The provided SD-OCT line scans demonstrate the largest horizontal extent of the tested cRORA lesion (orange) and in-eye control region (blue). In the magnification of the OCT scan, cRORA lesions extents are marked by the red arrowheads. The corresponding AOSLO images of both sites (cRORA vs. in-eye control) are presented. The cRORA lesions margins exceed the size of AOSLO imaging area.

For the precise localization of cRORA lesions, each B-scan of the 241-volume SD-OCT scan was assessed by two experienced medical expert graders (MS, LvDE) with specific expertise in early and late-stage AMD trials. The largest horizontal diameter of the lesion was annotated within the corresponding OCT B-scan. For MP testing, in-eye control regions were chosen by eccentricity matching via mirroring along the foveal vertical meridian. Control regions were allowed to present AMD-typical structural alterations but were not allowed to have any evidence for iRORA or cRORA lesions. In case these requirements were not met, the control region was shifted horizontally or vertically to a valid retinal region, which was found in all cases by shifting less than 100 µm from the initially targeted area. Per eye, a single cRORA lesion and a single in-eye control region were examined with clinical conventional MPand AOSLO-MP. cSLO and OCT images showing the MP target locations and the corresponding AOSLO images of both the cRORA and in-eye control region are presented in Figure 1.

### 2.4. Retinal Sensitivity Testing

#### 2.4.1. Conventional Clinical Microperimetry Testing

For the assessment of localized retinal sensitivities at retinal lesions in presence and absence of cRORA, study patients underwent conventional available clinical MP testing with individualized lesion-specific testing grids according to the topography position of the cRORA lesion with the Macular Integrity Assessment (S-MAIA; CenterVue, Padova, Italy) device (25 stimuli covering 2° of the central retina, 4–2 dB staircase strategy, Goldmann III [stimulus size: 0.43°, ~125 µm in an emmetropic eye], background apostilb (asb, or 1.3 cd/m^2^), dynamic threshold range 0–36 dB). Prior to each main examination, an initial training session was performed in all study participants using achromatic stimuli (400–800 nm) and a foveal centered testing grid. A patient-tailored MAIA grid stimulation pattern (5 × 5 stimulus points, spaced 0.5 deg) was crafted according to the exact topographic position of the cRORA lesion in the en-face infrared (IR) image of the SD-OCT scan using Matlab (version: Matlab2024b) and applied for localized sensitivity testing. Further, localized retinal sensitivity was assessed at an in-eye control region (in absence of both incomplete and complete RORA [7,8]) tested with an identical test grid.

#### 2.4.2. AOSLO-Based Imaging and Retinal Sensitivity Testing

A custom dual channel confocal AOSLO was employed to simultaneously image the retina (wavelength 840 nm; field of view: 0.85°) and to deliver visual stimuli at 543 nm (test spot diameter: 0.07°) [20]. The custom AOSLO has been comprehensively detailed previously [21,22]. Briefly, the system used a broadband laser source (SuperK EXTREME; NKT Photonics, Birkerod, Denmark) to provide multiple light channels. The integrated adaptive optics consisted of a Shack-Hartmann wavefront sensor (SHSCam AR-S-150-GE; Optocraft GmbH, Erlangen, Germany) and a deformable mirror (DM97-08; ALPAO, Montbonnot-Saint-Martin, France) in closed loop. The imaging wavelength was used for wavefront sensing. Two acousto-optic modulators (TEM-250-50-10-2FP; Brimrose, Sparks Glencoe, MD, USA) arranged in sequence produced high-contrast visual stimuli at 543 nm [20,23]. The 840 nm AOSLO imaging field generated a steady background illumination of 13.2 asb (4.2 cd/m^2^) against which the 543 nm test spots were presented. Visual stimuli could be displayed over a 50 dB dynamic range [20,23].

To stabilize the patient’s head, a dental impression stage (bite bar) was used. During imaging and testing, patients were instructed to maintain fixation on a small visual annulus, which was projected using a pellicle beam splitter. Sensitivity thresholds were measured using a 4–2 dB descending staircase method, requiring three threshold crossings to establish reliable values. Sensitivity thresholds at both cRORA and control sites were averaged from a minimum of five valid repeated runs at each location (5 tests at cRORA, 5 at control). Any runs that exhibited excessive catch or lapse trial errors, or those where either the patient or examiner noted any issues, were excluded from the final analysis. Prior to testing, AOSLO images were recorded at the respective retinal locations. To ensure familiarity with the procedure, patients underwent several test runs. While each AOSLO-MP test run took less than a minute, the full process, including AOSLO imaging and sensitivity testing, required approximately one hour per eye.

### 2.5. Statistical Analysis

A one-sided two-sample *t*-test was carried out using the *ttest2* Matlab function (version: Matlab2024b) to test for statistical significance (*p* < 0.05) of AOSLO-MP sensitivity loss in the presence and absence of cRORA. Furthermore, in a sub-analysis of the clinical MP examination, the retinal sensitivity threshold of the testing point at the center of the cRORA lesion was compared to the mean sensitivity values of the eight closed test stimuli at the margin of the corresponding cRORA lesion.

## 3. Results

**Figure 2:**
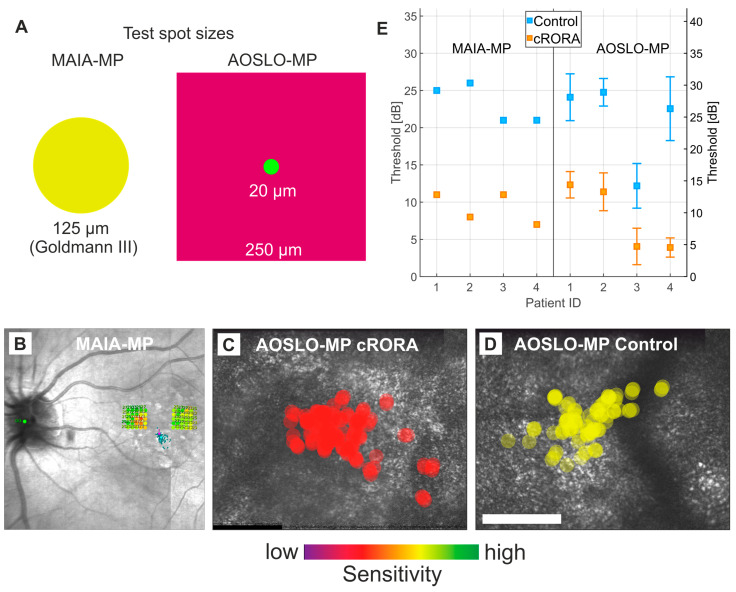
**Retinal sensitivity thresholds at cRORA lesions and in-eye control regions in iAMD.** (**A**) Spot size of the MAIA-MP (achromatic stimulus) and the AOSLO-MP (543 nm stimulus within the larger 840 nm AOSLO raster). (**B**) MAIA-MP testing grid and results of retinal sensitivity at the cRORA lesion (17 dB, red) and control region (27 dB, yellow) in patient #1 (for OCT correlation please consider patient 1 in Figure 1). The green dot highlights the optic disc control, the red cross the central fixation target and the blue dots the foveal fixation control. (**C**,**D**) AOSLO-MP test spot size and location at the cRORA lesion (**C**) and in-eye control region (**D**) in patient #1. AOSLO imaging shows patches of normal hexagonally arranged cone photoreceptors and areas of hyporeflective structural changes at both sites. (**E**) Mean sensitivity thresholds at the cRORA lesions and control regions for MAIA-MP and AOSLO-MP for all study patients. Note that both MP devices feature different background illumination and stimulus power range levels as indicated by the distinct left and right y-axis labels. Thus, sensitivity thresholds expressed as the inverse of stimulus power attenuation (in dB) are not directly comparable.

Four eyes of four intermediate to early late-stage AMD patients (mean age: 71.5 years, range: 70–75 years) were included. Comprehensive patient characteristics and localized sensitivity results for the tested retinal locations are provided in Table 1. Localized clinical MP testing revealed a mean retinal sensitivity of 9.3 ± 2.1 dB at cRORA lesions compared to 23.3 ± 2.2 dB at in-eye control regions, resulting in a significant mean sensitivity loss of −14 ± 3.3 dB, *p* < 0.01. Similarly, mean sensitivity thresholds tested by the AOSLO MP device were 9.3 ± 4.6 dB at cRORA lesions and 24.4 ± 6.9 dB at in-eye control regions leading to a significant overall mean sensitivity loss of −15.1 ± 5.1 dB, *p* < 0.01 (Figure 2). The test duration time of the main examination was about 5–8 min for the conventional and 60 to 90 min for the AOSLO- MP testing.

### 3.1. Retinal Sensitivity Thresholds at cRORA Margin

Considering clinical MP retinal sensitivity values at the margin of the cRORA, there was a mean sensitivity loss of −10.5 dB compared to the sensitivity value within the center of the cRORA lesion. Detailed results for each study patient are presented in Table 1.

#### Presentation of Cases

Patient #1 (f, 70 years) presented with hyporeflective patches at the photoreceptor level of the cRORA lesion surrounded by subtle hyperreflective material, while the in-eye control region exhibited an irregular photoreceptor mosaic with patches of slightly hypo- and hyperreflective photoreceptors (Figure 1). AOSLO-MP testing revealed a retinal sensitivity threshold of 14.4 dB at the cRORA and of 28.1 dB (13.7 dB difference, *p* < 0.01 Wilcoxon-Mann-Whitney test) at the in-eye control region. Retinal sensitivity differences to the in-eye control region in conventional clinical MP sensitivity were −14.0 dB compared to the center and −3.25 dB compared to the margin of the cRORA lesion.

Patient #2 (f, 70 years) presented with a comparable structure of the lesion on the SD-OCT scan as well as the photoreceptor mosaic in AOSLO imaging although there was a strong noticeable aggregation of hyperreflective material at the center of the cRORA lesion. Differences of retinal sensitivity thresholds between the control and lesion site though were still comparable to results in patient 1 with a sensitivity loss of −15.6 dB for AOSLO testing and −18.0 dB for the clinical MP at the center and −4.25 dB at the margin of the cRORA lesion.

Patient #3 (f, 75 years) and patient #4 (f, 71 years) displayed the picture of non-healthy patches of photoreceptors surrounded by subtle hyperreflective material and irregularly arranged photoreceptors at the corresponding cRORA location. The respective in-eye control region in both patients further gave evidence for a globally changed architecture of photoreceptors arrangement with additional presence of subretinal drusenoid deposits (SDDs) in patient 3. Interestingly, AOSLO-MP testing revealed an overall higher sensitivity loss between the cRORA and in-eye control region in patient 4 (−21.7 dB) compared to patient 3 (−9.5 dB), though the extent of functional impairment for clinical MP testing at the center and at the margin of the cRORA lesion was comparable between patients (for detailed results also compare Table 1).

## 4. Discussion

In this study, spatially resolved retinal sensitivity at cRORA lesions yielded significant and overall comparable sensitivity losses in both the conventional clinical available MP (−14.0 dB) and the experimental AOSLO-MP (−15.1 dB). High-resolution AOSLO imaging visualized the loss of the mosaic structure of single photoreceptor cells at cRORA lesions. In addition, our study demonstrated impaired retinal sensitivity at the margin of cRORA lesions with conventional clinical MP testing.

Across our study participants, structural biomarkers secondary to intermediate AMD presented with two different sensitivity thresholds:

(1) patients 1 and 2 displayed comparable sensitivity values with both MAIA-MP as well as AOSLO-MP, although retinal sensitivity values were lower at the cRORA lesion in patient 2. One possible reason could be the presence of hyperreflective material at the level of Bruch’s membrane at the cRORA lesion in patient 2. This material, also termed as refractile deposits, is a well-known high risk for a progressive visual decline and disease progression and therefore most likely attributed to a stronger localized impairment of retinal function [24,25]. Histological studies further described refractile deposits as part of drusen regression marked by the loss of RPE cells [26].
(2) in contrast to patients 1 and 2, in patients 3 and 4, we noticed a more global loss of photoreceptor function with an overall loss of retinal sensitivity at both retinal sites—at the cRORA lesion as well as at the control region. These patients presented with a more severe structurally affected retina, including thinning of the outer nuclear layer. In patient 3, multimodal retinal imaging displayed the concomitant presence of subretinal drusenoid deposits (SDD). SDD are known for an overall stronger impairment of photoreceptor function due to a global outer retinal thinning compared to patients presenting with conventional sub-RPE drusen alone [27,28,29]. In patient 4, there also was evidence for a more wide-spread affection of the retina with structural alterations being present at both the cRORA as well as the in-eye control region, in absence of SDD. In this context, previous studies have shown that thinning of the outer retinal layers precedes RPE loss in geographic atrophy [30,31,32].

Comparing our data with previous findings in eyes with iRORA lesions [17], sensitivity loss at cRORA lesions was nearly twice as high in conventional clinical MP testing with a mean sensitivity loss of −14 dB at cRORA lesions vs. −7.3 dB at iRORA lesions. However, it is important to note that besides the stage of GA formation at the test-lesions, there were further differences in the extent of detectable structural alterations as well as the topography of the tested in-eye control regions in both studies. These aspects must also be considered when comparing sensitivity values and especially sensitivity losses between study groups. This is most likely also explaining the AOSLO-MP testing results, revealing an overall higher mean sensitivity loss of −20.1 dB at iRORA lesions compared to −15.1 dB at cRORA lesions [17]. In this study, eyes with cRORA lesions were found to have overall lower retinal sensitivity with AOSLO-MP in the control region of the eye, resulting in an overall lower AOSLO-MP loss of sensitivity in this study.

Of note, although the patients with cRORA lesions demonstrated reduced average sensitivity, the depth of the sensitivity loss between study and control sites were comparable for patients 1 and 2 with cRORA of this study to patients 3 and 4 with iRORA from our previous publication [17]. The variability in retinal sensitivity in cRORA lesions detected here is supported by findings from Wu and coworkers showing that additional features of OCT imaging (presence of choroidal hypertransmission ≥ 500 µm, complete RPE loss ≥ 250 µm and manifestation of nascent GA ≥ 500 µm) are independently decisive for the depth of the functional loss [13].

The less severe retinal sensitivity loss at the margin of cRORA lesions indicates residual retinal function in spite of apparent structural alterations. In addition, Pfau and coworkers demonstrated the presence of retinal function at the direct margin of GA lesions (~125 µm) with smaller sensitivity losses at more distant test-points up to ~375 mm from the GA boundary [9]. Moreover, several groups showed evidence for the lack of an absolute functional scotoma (<0 dB) due to a remaining residual cone function within GA lesions, what is in line with our functional testing results within cRORA lesions using both the MAIA as well as the AOSLO based perimetry [33,34]. Therefore, slowing down further expansion of cRORA lesions might maintain the residual function and photoreceptors beyond the junctional zone of GA and can serve as a highly relevant structural endpoint for future interventional trials in AMD.

Major strengths of this study are the inclusion of highly motivated participants for a detailed and demanding psychophysical probing of localized retinal function at selected cRORA lesions. Furthermore, participants underwent detailed multimodal retinal imaging with the combined use of SD-OCT as well as high-resolution AO-imaging for analyzing structural alterations, allowing the assessment of individual photoreceptor cells. In addition, finely spaced testing grids, accurately aligned with the cRORA lesions were applied and compared to localized sensitivity values at sites within the same eye. A limitation of our study is the small number of patients examined. To prove our current clinical observations, validation in larger iAMD study cohorts, including assessment of medium time reproducibility of sensitivity measurements, is prudent. A further limitation is that in-eye control regions displayed varying degrees of AMD pathology, i.e., degenerative changes. An alternative approach could be to compare the sensitivity with exactly the same topographic retinal location from a healthy cohort, when such data becomes available. Additionally, both MP devices are fundamentally different regarding their technical specifications limiting their comparability. As previously noted, the AOSLO-MP background illumination is about 3.3 times brighter than the MAIA-MP’s background illumination and the stimulus colors are green and white, respectively [17]. Furthermore, the AOSLO-MP stimulus dynamic range is 50 dB compared to a 36 dB range of the MAIA-MP [21]. These differences result in an overall distinct device-specific subjective viewing situation while performing the MP tests.

To conclude, our study revealed a statistically significant severe impairment of localized sensitivity associated with cRORA lesions in patients with AMD, with a markedly reduced sensitivity but not complete loss of retinal function within as well as at the margin of cRORA lesions. This is one of the first studies employing both conventional clinical MP and experimental AOSLO perimetry for testing retinal sensitivity at early stages of atrophy development. These findings highlight the potential of cRORA lesions as a meaningful and functionally relevant clinical endpoint for future interventional trials in intermediate AMD.

## Figures and Tables

**Table 1:** **Study cohort characteristics and localized retinal sensitivity thresholds.** Retinal sensitivity at the margin of the cRORA lesion was only tested with the conventional clinical available MAIA microperimetry device.

						Mean MAIA-MP Thresholds [dB]			Mean AOSLO-MP Thresholds [dB]	
Patient	Sex	Age [Years]	Eye	Lens Status	BCVA [logMAR]	Stimulus at the Center of cRORA Lesion	Test Stimuli at Margin of cRORA Lesion	In-Eye Control	cRORA Lesion	In-Eye Control
#1	f	70	OS	Phakic	0.0	11	21.75	25	14.4	28.1
#2	f	70	OD	Pseudophakic	0.0	8	21.75	26	13.3	28.9
#3	f	75	OS	Phakic	0.2	11	17	21	4.7	14.2
#4	f	71	OD	Pseudophakic	0.1	7	18.5	21	4.6	26.3

## Data Availability

Data available upon reasonable request.

## Abbreviations

BCVA	best corrected visual acuity
logMAR	logarithm of the minimum angle of resolution
MP	microperimetry
dB	Decibel
AOSLO	adaptive optics scanning light ophthalmoscope
